# Association between Nightlife Goers’ Likelihood of an Alcohol Use Disorder and Their Preferred Bar’s Closing Time: A Cross-Sectional Observational Study in Perth, Australia

**DOI:** 10.3390/ijerph182413040

**Published:** 2021-12-10

**Authors:** William Gilmore, Martyn Symons, Wenbin Liang, Kathryn Graham, Kypros Kypri, Peter Miller, Tanya Chikritzhs

**Affiliations:** 1 National Drug Research Institute and enAble Institute, Faculty of Health Sciences, Curtin University, Perth, WA 6845, Australia; Martyn.Symons@curtin.edu.au (M.S.); T.N.Chikritzhs@curtin.edu.au (T.C.); 2 School of Public Health, Fujian Medical University, Fuzhou 350108, China; wenbin_liang@fjmu.edu.cn; 3 Institute for Mental Health Policy Research, Centre for Addiction and Mental Health, Toronto, ON M5S 2S1, Canada; kgraham@uwo.ca; 4 School of Medicine and Public Health, University of Newcastle, Callaghan, NSW 2308, Australia; kypros.kypri@newcastle.edu.au; 5 Centre for Drug use, Addictive and Anti-social Behaviour Research, Deakin University, Geelong, VIC 3220, Australia; peter.miller@deakin.edu.au

**Keywords:** nightlife-goers, bars, on-trade licensed outlets, trading hours, closing times, AUDIT-C, alcohol use disorders, alcohol policy

## Abstract

Introduction and aims: Associations between longer-term alcohol-related conditions and licensed outlet trading hours are not well understood. We investigated the association between nightlife-goers’ likelihood of an alcohol use disorder (AUD) and their preference for bars with special permits to remain open ‘late’ (i.e., spent more time there compared to any other venue) until 2 a.m. or 3 a.m. (Friday; Saturday) or midnight (Sunday) compared to bars with ‘standard’ closing times of midnight (Friday; Saturday) or 10 p.m. (Sunday). Design and methods: A cross-sectional observational study was conducted in four major nightlife areas of Perth, Australia, in 2015–2016. We conducted weekend street intercept surveys outside bars between 8 p.m. and 3 a.m. and screened participants who reported alcohol use prior to the survey and spent more time in a bar than any other venue type (*n* = 667) regarding their past year drinking pattern using AUDIT-C (*n* = 459). We used gender-specific logistic regression models to estimate associations between AUDIT-C categories (1–4, low risk; 5–7, hazardous; 8–12, active AUD) and preference for bars with different closing times (late vs. standard). Results: A large proportion of participants were hazardous drinkers or had active AUD (83% males; 65% females), and over half preferred a late to a standard closing bar. We found evidence of a positive association between preference for late closing bars and hazardous drinking females (OR = 3.48; 95% CI 1.47–8.23; *p* = 0.01), but not for females with active AUD, male hazardous drinkers, nor males with active AUD. Discussion and conclusions: Our study adds new evidence on associations between likelihood of AUD among nightlife-goers and trading hours. With increasing international relaxation of trading hours, evidence that late closing bars may be preferred by hazardous drinking females will be of concern to policymakers wanting to curb alcohol-related harms in the community.

## 1. Introduction

In countries where alcohol is a legal and regulated product, government control over availability is most frequently exercised through taxation, minimum legal purchase age, and a licensing system for production, wholesale and retail—regulating how, when and where outlets operate. Decades of accumulated international research, predominantly from North America, Northern Europe and Australasia, have identified that restrictions on alcohol’s economic (i.e., retail price relative to disposable income) and physical (e.g., numbers of outlets, trading hours) availability are key to effectively reducing population-level alcohol consumption and related harms [1].

Availability theory suggests that greater availability will affect harm by affecting the distribution of drinking behaviors, and those harms will differ by population subgroups according to their drinking patterns and behaviors [2]. In response to changes in availability, changes in underlying drinking patterns in a population may lead to changes across a range of alcohol-related harms. For instance, research distinguishes between heavy episodic drinking that results in increased risk of shorter-term harms (e.g., injury from road traffic crashes and violence) and regular heavy use that results in increased risk of longer-term harms (e.g., alcohol dependence and liver cirrhosis) [3].

Studies of alcohol availability effects, particularly physical availability, such as outlet density and trading hours, have tended to focus on shorter-term harms (e.g., assault). By comparison, physical availability effects on potential longer-term harms, such as risk of alcohol use disorders (AUD), have been less well explored. In Australia, for instance, only two outlet density studies have examined longer-term outcomes. A longitudinal study from Victoria found off-trade outlet density was positively associated with hospitalization rates for longer-term alcohol-related conditions [4], and a cross-sectional study from Western Australia found patients’ residential proximity to off-trade outlets was associated with increased risk of secondary care contact for anxiety, stress and depression [5].

Systematic reviews have generally concluded that even relatively small extensions or restrictions applied to on-trade (where alcohol is consumed *on* the premises e.g., bars, nightclubs) or off-trade (where alcohol is consumed elsewhere, e.g., liquor stores, supermarkets) outlet trading hours can change population-level alcohol consumption and related harms [6,7,8,9,10,11,12]. Moreover, a meta-analysis of six natural experiments that investigated associations between off-trade days of alcohol sale and per capita consumption from North America and Sweden, found an additional day of alcohol sale was associated with a 3.4% increase in per capita consumption [13].

There are few studies of the associations between longer-term alcohol-related problems and licensed outlet trading hours. One German controlled interrupted time series analyses evaluating a state’s ban on alcohol sales from off-trade outlets between 10 p.m. and 5 a.m. found reductions in hospitalizations for mental and behavioral disorders due to use of alcohol (ICD10 code: F10; includes acute intoxication, harmful use and dependence) in both male and female adolescents and young adults in the post intervention period, though the effect on males was stronger [14]. To our knowledge, only one study has investigated the association between likelihood of AUD and alcohol outlet trading hours. Conducted in Perth, Australia, almost 40 years ago, the study compared drinkers at bars opening at 6 a.m. or 7 a.m. with drinkers at bars opening later at 10 a.m. Using an abbreviated form of the Michigan Alcoholism Screening Test, the study found that males who drank at early opening pubs were more likely to obtain scores indicative of problem drinking compared to males who drank at later opening bars [15].

Despite a narrowing gap between males and females in terms of their levels of alcohol consumption in Australia, there are still marked differences in their drinking patterns from national surveys of the general population [16] and from national surveys of nightlife-goers [17]. Therefore, it is important to consider possible gender differences in analyses of availability and alcohol use. We investigated the association between likelihood of AUD among nightlife-goers who went ‘out’ drinking in Perth, Australia, and their preference for bars with different closing times (late vs. standard; spent more time in a late or standard bar compared to any other venue). We hypothesized that those with a drinking pattern indicating hazardous use or active AUD would be more likely to prefer bars with late closing hours to standard closing bars compared to low risk drinkers, and that there would be difference between males and females for these associations. To our knowledge, this is the first nightlife study to investigate whether past year alcohol consumption patterns among nightlife-goers are associated with the trading hours of their preferred bars.

## 2. Methods

### 2.1. Street Intercept Surveys

Trained teams of between six and 12 researchers conducted street intercept surveys from November 2015 to April 2016 in metropolitan Perth’s four main nightlife precincts: Perth City (five sessions); Northbridge (five sessions); Leederville (two sessions); Fremantle (one session). We avoided major events and public holidays when atypical drinking sessions may occur (e.g., New Year’s Eve, Australia Day). Surveys took place in public spaces between 8 p.m. and 3 a.m. on either a Friday (five sessions) or Saturday (six sessions) and between 8 p.m. and midnight on a Sunday (two sessions). To approximate a random sample, field workers invited every third person who walked past them to participate. Field workers recorded non-responses as declines to participate only after a person had engaged with them and had the purpose of the study explained to them. Overall, we achieved a response rate of 89%. Several studies of substance use in nightlife areas have employed a street intercept approach, for example, [18,19], and it has been shown to be effective in recruiting samples of nightlife-goers [20]. We selected survey locations strategically using DLGSCI information, bar websites and Google Maps™ to ensure gender-specific minimum quotas of 200 each for nightlife-goers preferring a late or standard closing bar.

Field workers delivered the survey instrument using Tap Forms™ on smartphones which automatically recorded date and time of survey. After gaining informed consent, participants self-reported gender, birth year and usual occupation. Field workers then asked participants a series of questions related to their drinking behaviors that night prior to survey including: Had they drunk any alcohol? How long had they been drinking? Had they been drinking at licensed venues? Had they been drinking elsewhere prior to drinking at licensed venues (i.e., pre-drinking)? Had they drunk energy drinks? Was it a typical night out for them?

If participants had been drinking at licensed venues, field workers asked them the names of the venues they had attended and about how much time had they spent at each. As described below, we used this question to define whether their preferred bar’s hours were late or standard closing. Field workers then asked participants the three Alcohol Use Disorder Identification Test—Consumption (AUDIT-C) questions, assessing frequency of drinking, typical number of drinks consumed on a drinking occasion, and frequency of six or more standard drinks, all over the past year [21]. AUDIT-C is a quick, simple, reliable and well validated tool to screen for hazardous drinking or active AUD based on past year drinking pattern [22,23,24,25] and has been used in research studies outside of clinical settings previously, for example, [26].

### 2.2. Bar’s Closing Times

The Perth liquor licensing system allows bars to apply to the Department of Local Government, Sport and Cultural Industries (DLGSCI) for extended trading permits that enable late night (or early morning) alcohol sales. At the time of the current study, standard closing for bars was midnight Monday to Saturday and 10 p.m. Sunday. However, after application and approval for a closing time extension, some were permitted to trade up until 2 a.m. or 3 a.m. Monday to Saturday and to midnight Sunday (late closing).

At the commencement of data collection, legislation change in local liquor licensing was implemented at very short notice [27]. From 20 November 2015, all bars (i.e., not just those with special permits) were allowed to trade up to midnight on Sunday nights rather than closing at 10 p.m. Surveys took place on two Sunday nights in the early months of the study (22 November, 20 December), and because we observed little uptake of these relaxed trading hours on the ground we did not change what constituted late (midnight) vs. standard (10 p.m.) closing bars for these dates. We halted Sunday surveys at the end of 2015 due to the potential for bars to start taking up the newly relaxed trading hours and because it was more difficult to meet survey quotas on quieter Sunday nights.

### 2.3. Survey Data

Regardless of the time of survey, participants who reported alcohol use may not have visited a licensed venue at all or may have visited a number of different venues on their night out (including restaurants, both standard and late closing bars, nightclubs etc.). Participants surveyed past midnight, therefore, were not necessarily drinkers from late closing bars and vice versa. We defined participants’ ‘preferred venue’ as where they had spent more time that night compared to other venues and assumed this is where they had probably consumed most alcohol. We coded ‘preferred bars’ according to whether they had standard (0) or late (1) closing using DLGSCI records and cross-checked against bar websites for currency. We found only one bar had its late trading permit revoked during the study period; in this instance bar trading status (late; standard) was coded based on date of survey and date of permit revocation.

We categorized AUDIT-C scores into three groups using the same raw score cut-offs for males and females: 1–4, low risk drinker; 5–7, hazardous drinker; 8–12, drinker with active AUD [28]. We estimated participant age using date of survey and year of birth then categorized into four groups: 18–21; 22–25; 26–29; ≥30. We classified occupation according to the Australian and New Zealand Standard Classification of Occupations (plus an ‘Other’ category to capture students, stay-at-home parents, unemployed etc.) [29] and grouped as follows: manager/professional; technician/trade/laborer; community/personal service; clerical/administrative/sales; other. We dichotomized time of survey into ‘before midnight’ and ‘midnight and after’, reflecting the distinction between late and standard closing bars. In order to reflect typical night-time drinking occasions, we categorized day of survey (i.e., Friday, Saturday or Sunday) according to when data collection sessions were initiated, for example, surveys undertaken between 10 p.m. Friday night and 2 a.m. the following morning were all considered a ‘Friday’ night survey.

### 2.4. Statistical Analysis

We used Pearson’s chi-square tests and independent samples t-tests to explore bivariate associations (Table 1). We used multivariable logistic regression models to investigate whether participant likelihood of AUD was associated with preferring a standard or late closing bar (Table 2). We ran two gender-specific models and adjusted for a range of potential confounders including: age, occupation, day of survey, time of survey, drinking session duration, whether it was a typical night out, pre-drinking and energy drink use using a backward stepwise selection approach. Hosmer and Lemeshow statistics assessed the models’ goodness-of-fit. We used SPSS Statistics v27.0 (IBM Corp, Armonk, NY, USA) for all analyses [30].

### 2.5. Ethics

We conducted this study in accordance with the National Statement on Ethical Conduct in Human Research and the Human Research Ethics Committees at Curtin University approved it (HR154/2015). Participants provided informed consent to field workers who recorded responses in an electronic data collection smartphone application.

## 3. Results

As shown in Table 1, of the 667 participants who reported alcohol use at a licensed venue and preferred a bar to other venue types, 459 completed the AUDIT-C. A large proportion of participants were hazardous drinkers (40% males; 45% females) or had active AUD (42% males; 20% females). Over half of male and female participants preferred a late closing bar to a standard closing bar. Gender-specific bivariate analyses indicated evidence of association between AUDIT-C and bar closing time for females but not males. Hazardous drinking females preferred late closing bars over standard closing bars, but for low risk drinking females and females with active AUD the association was the opposite. Age was associated with preferred bar for female participants, with those in all age groups except 26–29 more likely to prefer later closing. Participants were from a range of occupations, but there was no evidence of association between occupation and bar preference. For males and females, pre-drinking was more common among those preferring late closing bars. Less than a fifth of participants reported energy drink use, with males who used energy drinks more likely to prefer late closing bars. Half reported that it was not a typical night out for them, with males reporting a non-typical night out more likely to prefer late closing bars. In terms of survey characteristics, preference for late closing bars was more likely among those surveyed on Friday nights than on Saturday nights and more likely among those surveyed after midnight than before midnight.

Hosmer and Lemeshow statistics raised no concerns about the goodness-of-fit of the two logistic regression models (Table 2). Model results indicated no evidence of association between males’ AUDIT-C category and their preferred bar’s closing time. For male participants, the preference for late-closing bars was associated with the following: the youngest age group (age 18–21); clerical occupations (compared to ‘other’); and the survey occurring on Friday night.

Model results for female participants indicated an association between a preference for late closing bars and hazardous drinking (OR = 3.48; 95% CI 1.47–8.23; *p* = 0.01) compared to low risk drinking, but not for active AUD. For females, there was also a positive association between a preference for late closing bars and being surveyed on Friday night compared to Saturday night, but a negative association with being 26–29 years old compared to 30 years and older.

## 4. Discussion

Female hazardous drinkers were more likely to prefer a late closing bar when compared to female low risk drinkers. We found no evidence to support our hypothesis of a positive association between preference for late closing bars and females with active AUD, male hazardous drinkers or males with active AUD.

International research has demonstrated positive associations between licensed outlet trading hours and population-level per capita alcohol consumption [13], and both male and female adolescent and young adult hospitalizations for mental and behavioral disorders due to use of alcohol (encompassing shorter-term and longer-term harms) [14]. The only study that we are aware of that has focused specifically on the relationship between AUD and patron attendance at bars with extended trading hours found a positive association for males (females were not included in the study) [15]. However, that study investigated the association for *earlier* opening hours rather than *later* closing as in the present study. Our study was partly consistent with those findings but only for female hazardous drinkers (not for the heaviest drinking females nor male participants at all). Sample size for females with active AUD was smaller than for the other AUDIT-C categories and this may have affected statistical power. Gender differences in our findings may be related to other characteristics of bars themselves that we were unable to adjust for, for example, in terms of their target audience, marketing and entertainment. The lack of evidence for association for males may also be explained by the high proportion who said it was not a typical night out. Thus, male attendance (or lack of attendance) at a late closing bar on the night of survey may have been less reflective of their usual pattern.

Licensed outlets’ closing times and their associations with harm are a policy issue highly relevant to liquor licensing, health and law enforcement authorities and to the general public.

Our results are directly relevant for Western Australian decision makers in the wake of state-wide Sunday closing time relaxation (from 10 pm to midnight) for bars in 2015 (see methods) and in light of proposals to introduce Sunday trading for liquor stores in regional areas across the state (currently restricted except for cases where extended trading time permits are held), both recommendations coming out of a review of the Western Australian Liquor Control Act in 2013 [26]. At present, applications and decisions relating to extended trading time permits for bars are made by the Department of Local Government, Sport and Cultural Industries on an ad hoc basis, likely with inconsistent reference to research evidence. As international and even national research findings can often be interpreted as unrelated to local contexts, this study may help to fill a local knowledge gap. As well, it may suggest more generally an important link between later closing and hazardous drinking among females.

### Limitations

We made several assumptions in assigning participants to late vs. standard closing bars. We assumed that time spent in a bar was positively associated with quantity of alcohol consumed, which may not necessarily be the case. We also assumed spending most time in one venue type meant that the sum of time over the night would be in favor of that venue type, that is, participants who spent two hours in one late closing bar and one hour in each of three standard closing bars will have been assigned as preferring late closing bars. Furthermore, half of participants were not on a typical night out for them so may have been drinking at venues they did not typically frequent and/or may have gone home earlier or stayed out later than usual.

It is important to note that we were only able to discern evidence of cross-sectional associations between nightlife-goers’ heavy drinking and their preference for late closing bars not whether heavier drinking leads to frequenting later closing bars or vice versa. The study also used self-report which may not be the most accurate measure as cognitive ability declines with alcohol intoxication [31]. Finally, our results may not be generalizable to other nightlife areas outside of Perth, Australia.

## 5. Conclusions

Our study adds new evidence to the alcohol physical availability research on associations between longer-term alcohol problems among nightlife-goers and alcohol outlet trading hours. With increasing state, national and international relaxation of trading hours for licensed outlets, evidence that preference for later closing bars is associated with hazardous drinking among females will be of concern to policymakers wanting to curb alcohol-related harms in the community.

## Figures and Tables

**Table 1:** Gender-specific descriptive statistics and bivariate analyses for participant and survey characteristics by participants’ preferred bar’s closing time.

Variables ^±^	Male					Female				
	Late		Standard			Late		Standard		
Participant characteristics	*n*	%	*n*	%		*n*	%	*n*	%	
AUDIT-C					*χ*^2^(2) = 1.2, *p* = 0.54					*χ*^2^(2) = 10.2, *p* = 0.01
1–4 (low risk)	27	16	27	19		23	27	29	46	
5–7 (hazardous)	67	39	58	41		48	56	19	30	
8–12 (active AUD)	77	45	55	39		14	16	15	24	
Total	171	100	140	100		85	100	63	100	
Age					*χ*^2^(3) = 7.0, *p* = 0.07					*χ*^2^(2) = 8.2, *p* = 0.04
18–21	46	18	24	12		39	31	19	22	
22–25	73	29	48	24		41	32	25	29	
26–29	56	22	59	29		16	13	24	28	
≥30	76	30	70	35		31	24	18	21	
Total	251	100	201	100		127	100	86	100	
Occupation					*χ*^2^(4) = 8.4, *p* = 0.08					*χ*^2^(4) = 3.9, *p* = 0.42
Manager/professional	83	34	77	39		29	24	28	33	
Technician/trade/labourer	88	36	65	33		8	7	6	7	
Community/personal service	18	7	15	8		25	20	12	14	
Clerical/administrative/sales	24	10	7	4		28	23	22	26	
Other	31	13	33	17		33	27	17	20	
Total	244	100	197	100		123	100	85	100	
Pre-drinking					*χ*^2^(1) = 3.8, *p* = 0.05					*χ*^2^(1) = 8.8, *p* < 0.01
No	110	44	108	53		52	41	53	62	
Yes	140	56	95	47		75	59	33	38	
Total	250	100	203	100		127	100	86	100	
Energy drink use					*χ*^2^(1) = 8.3, *p* < 0.01					*χ*^2^(1) = 1.4, *p* = 0.24
No	205	82	185	91		110	87	79	92	
Yes	46	18	18	9		17	13	7	8	
Total	251	100	203	100		127	100	86	100	
Was it a typical night out?					*χ*^2^(2) = 2.0, *p* = 0.37					*χ*^2^(2) = 2.9, *p* = 0.24
No, usually smaller	44	33	32	25		20	25	16	27	
No, usually bigger	28	21	27	21		13	16	16	27	
Yes	62	46	68	54		46	58	27	46	
Total	134	100	127	100		79	100	59	100	
Drinking session duration (hours)	*n*	Mean (SD)	*n*	Mean (SD)		*n*	Mean (SD)	*n*	Mean (SD)	
	246	4.8 (2.7)	198	5.0 (2.5)	t (442) = 0.9, *p* = 0.31	126	4.5 (2.3)	86	4.4 (2.0)	t(210) = −0.3, *p* = 0.48
Survey characteristics	*n*	%	*n*	%		*n*	%	*n*	%	
Day					*χ*^2^(2) = 21.0, *p* < 0.001					*χ*^2^(2) = 10.1, *p* = 0.01
Friday	108	43	48	24		52	41	19	22	
Saturday	119	47	118	58		64	50	62	72	
Sunday	24	10	37	18		11	9	5	6	
Total	251	100	203	100		127	100	86	100	
Time					*χ*^2^(1) = 10.9, *p* = 0.001					*χ*^2^(1) = 3.6, *p* = 0.06
Before midnight	127	51	134	66		60	47	52	60	
Midnight and after	124	49	69	34		67	53	34	40	
Total	251	100	203	100		127	100	86	100	

^±^ Small or big night out are colloquialisms regarding level of perceived intoxication.

**Table 2:** Results from two gender-specific logistic regression models: Association between AUDIT-C category and participants’ preferred bar’s closing time (late = 1; standard = 0) adjusting for survey and participant characteristics ^±^.

Variables ^±^	Male (*n* = 306)					Female (*n* = 148)				
Participant characteristics	*n*	OR	LCI	UCI	*p*-Value	*n*	OR	LCI	UCI	*p*-Value
AUDIT-C										
1–4 (low risk) [Ref]	54					52				
5–7 (hazardous)	121	1.06	0.54	2.09	0.87	67	3.48	1.47	8.23	<0.01
8–12 (active AUD)	131	1.31	0.66	2.62	0.44	29	1.23	0.43	3.52	0.70
Age										
18–21	57	2.82	1.26	6.33	0.01	39	0.96	0.33	2.78	0.94
22–25	84	1.48	0.78	2.81	0.23	51	0.73	0.26	2.06	0.55
26–29	76	1.09	0.57	2.08	0.80	25	0.13	0.04	0.49	<0.01
≥30 [Ref]	89					33				
Occupation										
Manager/professional	100	2.11	0.96	4.65	0.07					
Technician/trade/labourer	115	2.02	0.96	4.25	0.06					
Community/personal service	20	1.22	0.41	3.62	0.72					
Clerical/administrative/sales	21	3.46	1.09	10.94	0.03					
Other [Ref]	50									
Survey characteristics										
Day										
Friday	111	1.92	1.14	3.22	0.01	53	3.22	1.43	7.26	<0.01
Saturday [Ref]	163					86				
Sunday	32	0.58	0.26	1.28	0.18	9	2.99	0.60	15.04	0.18

Male model: Hosmer and Lemeshow χ2(8) = 10.3, *p* = 0.25. Female model: Hosmer and Lemeshow χ2(7) = 1.1, *p* = 0.99. OR: Odds ratio. L/UCI: 95% lower/upper confidence interval. [Ref]: Reference group. ^±^ Time of survey, duration of drinking session, pre-drinking, energy drink use and whether it was a typical night out were non-contributing variables in both models and removed in the backward stepwise selection approach. Occupation was a non-contributing variable in the female model and was removed in the backward stepwise selection approach.

## Data Availability

The data presented in this study are available on request from the corresponding author subject to ethical approval.
