# Correction: Gilmore et al. Association between Nightlife Goers’ Likelihood of an Alcohol Use Disorder and Their Preferred Bar’s Closing Time: A Cross-Sectional Observational Study in Perth, Australia. *Int. J. Environ. Res. Public Health* 2021, *18*, 13040

**DOI:** 10.3390/ijerph19159684

**Published:** 2022-08-05

**Authors:** William Gilmore, Martyn Symons, Wenbin Liang, Kathryn Graham, Kypros Kypri, Peter Miller, Tanya Chikritzhs

**Affiliations:** 1 National Drug Research Institute and enAble Institute, Faculty of Health Sciences, Curtin University, Perth, WA 6845, Australia; 2 School of Public Health, Fujian Medical University, Fuzhou 350108, China; 3 Institute for Mental Health Policy Research, Centre for Addiction and Mental Health, Toronto, ON M5S 2S1, Canada; 4 School of Medicine and Public Health, University of Newcastle, Callaghan, NSW 2308, Australia; 5 Centre for Drug Use, Addictive and Anti-Social Behaviour Research, Deakin University, Geelong, VIC 3220, Australia

## Error in Table

In the original publication [1], there were mistakes in Table 1 and Table 2 as published. We had made a coding error with the independent variable “Was it a typical night out?”. The corrected Table 1 and Table 2 appear below.

## Text Correction

There was an error in the original publication [1]. We had made a coding error with the independent variable “Was it a typical night out?”, thereby affecting some of the text. Corrections have been made to the following sections:

Section 3, Paragraph 1:

Half reported that it was not a typical night out for them.

Section 3, Paragraph 2:

For male participants, the preference for late-closing bars was associated with the following: the youngest age group (age 18–21); clerical occupations (compared to ‘other’); and the survey occurring on Friday night.

Section 4, Paragraph 5:

Furthermore, half of participants were not on a typical night out for them so may have been drinking at venues they did not typically frequent and/or may have gone home earlier or stayed out later than usual.

The authors apologise for any inconvenience caused and state that the scientific conclusions are unaffected. This correction was approved by the Academic Editor. The original publication has also been updated.

## Figures and Tables

**Table 1:** Gender-specific descriptive statistics and bivariate analyses for participant and survey characteristics by participants’ preferred bar’s closing time.

Variables ^±^	Male					Female				
	Late		Standard			Late		Standard		
Participant characteristics	*n*	%	*n*	%		*n*	%	*n*	%	
AUDIT-C					*χ*^2^(2) = 1.2, *p* = 0.54					*χ*^2^(2) = 10.2, *p* = 0.01
1–4 (low risk)	27	16	27	19		23	27	29	46	
5–7 (hazardous)	67	39	58	41		48	56	19	30	
8–12 (active AUD)	77	45	55	39		14	16	15	24	
Total	171	100	140	100		85	100	63	100	
Age					*χ*^2^(3) = 7.0, *p* = 0.07					*χ*^2^(2) = 8.2, *p* = 0.04
18–21	46	18	24	12		39	31	19	22	
22–25	73	29	48	24		41	32	25	29	
26–29	56	22	59	29		16	13	24	28	
≥30	76	30	70	35		31	24	18	21	
Total	251	100	201	100		127	100	86	100	
Occupation					*χ*^2^(4) = 8.4, *p* = 0.08					*χ*^2^(4) = 3.9, *p* = 0.42
Manager/professional	83	34	77	39		29	24	28	33	
Technician/trade/labourer	88	36	65	33		8	7	6	7	
Community/personal service	18	7	15	8		25	20	12	14	
Clerical/administrative/sales	24	10	7	4		28	23	22	26	
Other	31	13	33	17		33	27	17	20	
Total	244	100	197	100		123	100	85	100	
Pre-drinking					*χ*^2^(1) = 3.8, *p* = 0.05					*χ*^2^(1) = 8.8, *p* < 0.01
No	110	44	108	53		52	41	53	62	
Yes	140	56	95	47		75	59	33	38	
Total	250	100	203	100		127	100	86	100	
Energy drink use					*χ*^2^(1) = 8.3, *p* < 0.01					*χ*^2^(1) = 1.4, *p* = 0.24
No	205	82	185	91		110	87	79	92	
Yes	46	18	18	9		17	13	7	8	
Total	251	100	203	100		127	100	86	100	
Was it a typical night out?					*χ*^2^(2) = 2.0, *p* = 0.37					*χ*^2^(2) = 2.9, *p* = 0.24
No, usually smaller	44	33	32	25		20	25	16	27	
No, usually bigger	28	21	27	21		13	16	16	27	
Yes	62	46	68	54		46	58	27	46	
Total	134	100	127	100		79	100	59	100	
Drinking session duration (hours)	*n*	Mean (SD)	*n*	Mean (SD)		*n*	Mean (SD)	*n*	Mean (SD)	
	246	4.8 (2.7)	198	5.0 (2.5)	t (442) = 0.9, *p* = 0.31	126	4.5 (2.3)	86	4.4 (2.0)	t(210) = −0.3, *p* = 0.48
Survey characteristics	*n*	%	*n*	%		*n*	%	*n*	%	
Day					*χ*^2^(2) = 21.0, *p* < 0.001					*χ*^2^(2) = 10.1, *p* = 0.01
Friday	108	43	48	24		52	41	19	22	
Saturday	119	47	118	58		64	50	62	72	
Sunday	24	10	37	18		11	9	5	6	
Total	251	100	203	100		127	100	86	100	
Time					*χ*^2^(1) = 10.9, *p* = 0.001					*χ*^2^(1) = 3.6, *p* = 0.06
Before midnight	127	51	134	66		60	47	52	60	
Midnight and after	124	49	69	34		67	53	34	40	
Total	251	100	203	100		127	100	86	100	

^±^ Small or big night out are colloquialisms regarding level of perceived intoxication.

**Table 2:** Results from two gender-specific logistic regression models: Association between AUDIT-C category and participants’ preferred bar’s closing time (late = 1; standard = 0) adjusting for survey and participant characteristics **^±^**.

Variables ^±^	Male (*n* = 306)					Female (*n* = 148)				
Participant characteristics	*n*	OR	LCI	UCI	*p*-Value	*n*	OR	LCI	UCI	*p*-Value
AUDIT-C										
1–4 (low risk) [Ref]	54					52				
5–7 (hazardous)	121	1.06	0.54	2.09	0.87	67	3.48	1.47	8.23	<0.01
8–12 (active AUD)	131	1.31	0.66	2.62	0.44	29	1.23	0.43	3.52	0.70
Age										
18–21	57	2.82	1.26	6.33	0.01	39	0.96	0.33	2.78	0.94
22–25	84	1.48	0.78	2.81	0.23	51	0.73	0.26	2.06	0.55
26–29	76	1.09	0.57	2.08	0.80	25	0.13	0.04	0.49	<0.01
≥30 [Ref]	89					33				
Occupation										
Manager/professional	100	2.11	0.96	4.65	0.07					
Technician/trade/labourer	115	2.02	0.96	4.25	0.06					
Community/personal service	20	1.22	0.41	3.62	0.72					
Clerical/administrative/sales	21	3.46	1.09	10.94	0.03					
Other [Ref]	50									
Survey characteristics										
Day										
Friday	111	1.92	1.14	3.22	0.01	53	3.22	1.43	7.26	<0.01
Saturday [Ref]	163					86				
Sunday	32	0.58	0.26	1.28	0.18	9	2.99	0.60	15.04	0.18

Male model: Hosmer and Lemeshow χ2(8) = 10.3, *p* = 0.25. Female model: Hosmer and Lemeshow χ2(7) = 1.1, *p* = 0.99. OR: Odds ratio. L/UCI: 95% lower/upper confidence interval. [Ref]: Reference group. ^±^ Time of survey, duration of drinking session, pre-drinking, energy drink use and whether it was a typical night out were non-contributing variables in both models and removed in the backward stepwise selection approach. Occupation was a non-contributing variable in the female model and was removed in the backward stepwise selection approach.
